# Characterization of Differences in Chemical Profiles and Antioxidant Activities of *Schisandra chinensis* and *Schisandra sphenanthera* Based on Multi-Technique Data Fusion

**DOI:** 10.3390/molecules29204865

**Published:** 2024-10-14

**Authors:** Lujie Lin, Zhuqian Tang, Huijuan Xie, Lixin Yang, Bin Yang, Hua Li

**Affiliations:** Institute of Chinese Materia Medica, China Academy of Chinese Medical Sciences, Beijing 100700, China; lin5674qq@126.com (L.L.); tangzhuqian@163.com (Z.T.); xiehuijuann@163.com (H.X.); lxyang@icmm.ac.cn (L.Y.)

**Keywords:** *Schisandra chinensis*, *Schisandra sphenanthera*, chemical profile, antioxidant activity

## Abstract

*Schisandra chinensis* (Turcz.) Baill. (*S. chinensis*) and *Schisandra sphenanthera* Rehd. et Wils (*S. sphenanthera*) are called “Wuweizi” in traditional Chinese medicine, and they have distinct clinical applications. To systematically compare the differential characteristics of *S. chinensis* and *S. sphenanthera*, this study employed ultra-performance liquid chromatography–quadrupole time-of-flight mass spectrometry (UPLC-QTOF-MS) and gas chromatography–mass spectrometry (GC-MS) to construct chemical profiles of these two species from different regions. In total, 31 non-volatiles and 37 volatiles were identified in *S. chinensis*, whereas 40 non-volatiles and 34 volatiles were detected in *S. sphenanthera*. A multivariate statistical analysis showed that the non-volatiles tigloygomisin P, schisandrol A, schisantherin C, and 6-O-benzoylgomisin O and the volatiles ylangene, γ-muurolene, and β-pinene distinguish these species. Additionally, the metabolism of oxygen free radicals can contribute to the development of various diseases, including cardiovascular and neurodegenerative diseases. Therefore, antioxidant activities were evaluated using 1,1-diphenyl-2-picrylhydrazyl (DPPH) and 2,2′-azino-bis-3-ethylbenzthiazoline-6-sulphonic acid (ABTS) scavenging assays. The results showed that *S. sphenanthera* exhibited significantly higher antioxidant potential. A gray relational analysis indicated that the key contributors to the antioxidant activity of *S. chinensis* were schisandrol A, gomisin G, schisantherin C, pregomisin, gomisin J, and schisantherin B. For *S. sphenanthera*, the key contributors included gomisin K2, schisantherin B, gomisin J, pregomisin, schisantherin C, schisandrin, gomisin G, schisantherin A, schisanhenol, and α-pinene. The identification of the differential chemical markers and the evaluation of the antioxidant activities provide a foundation for further research into the therapeutic applications of these species. This innovative study provides a robust framework for the quality control and therapeutic application of *S. chinensis* and *S. sphenanthera*, offering new insights into their medicinal potential.

## 1. Introduction

Fructus Schisandrae is known as “bei wuweizi” and “nan wuweizi”, which refer to the dried mature fruits of *Schisandra chinensis* (Turcz.) Baill. and *Schisandra sphenanthera* Rehd. et Wils, respectively. These fruits have been used medicinally in China for thousands of years. According to the 2020 edition of the Chinese Pharmacopoeia, both species have the effects of “astringing and tonifying Qi, generating fluid, nourishing kidney and calming the heart” [1]. Chemically, *S. chinensis* and *S. sphenanthera* comprise both volatile and non-volatile components. The non-volatile components include lignans, organic acids, and polysaccharides (approximately 8%, 9%, and 12%, respectively), while the volatile components constitute about 6% of the total [2]. Dibenzocyclooctadiene lignans are the major active constituents in both species. They are known for their anti-inflammatory [3,4], antioxidant [5], and hepatoprotective activities [6].

Due to their morphological similarities and homogenous traditional applications, *S. chinensis* and *S. sphenanthera* are often confused in clinical practice. Chemical composition serves as a critical indicator of herbal medicine quality. Chemical profiling involves the use of chromatograms and spectrograms obtained through analytical methods such as HPLC-MS, UPLC-MS, GC-MS, etc., offering high sensitivity and broad selectivity to determine detailed chemical information related to herbs and food. Principal component analysis (PCA) and orthogonal partial least squares discriminant analysis (OPLS-DA) are commonly used to classify varieties of Chinese medicines and food products. Han et al. employed UPLC-Q-TOF/MS coupled with chemometrics to analyze the metabolic profiles and chemical differences of cultivated and wild types of Flos Chrysanthemi Indici. They identified five characteristic components that differentiate these two groups [7]. Guo et al. conducted comprehensive profiling of volatile and non-volatile components of both *S. chinensis* and *S. sphenanthera*, followed by data processing, which resulted in the identification of three lignans and seven volatile compounds as distinguishing markers [8].

Apart from chemical composition, biological activity is another crucial indicator of herbal medicine quality. A spectrum–effect relationship involves linking chemical information from chromatographic profiles to pharmacological outcomes through appropriate data processing methods, thereby achieving a comprehensive evaluation of quality and efficacy [9]. Previous studies have demonstrated a close association between free radicals and human diseases, and antioxidants can mitigate inflammation and oxidative stress, delaying aging by scavenging free radicals [10,11]. Despite being excellent sources of natural antioxidants, few studies have further explored the constituents associated with antioxidant activity in *S. chinensis* and *S. sphenanthera*. Pi et al. evaluated the relationships between the major lignans (schisandrol A, schisandrol B, schizandrin A, and schizandrin B) in *S. chinensis* and their antioxidant activities [12]. To date, no studies have conducted simultaneous comparisons of the chemical constituents and antioxidant activities of *S. chinensis* and *S. sphenanthera* while also identifying components closely associated with antioxidant activity.

To systematically analyze the constituent factors underlying the antioxidant activities of *S. chinensis* and *S. sphenanthera*, we initially established comprehensive chemical profiles of volatile and non-volatile components from different regions using UPLC-Q/TOF-MS and GC-MS techniques. Data fusion was employed to integrate results from different analytical instruments, complementing the chemical information and overcoming the limitations of single-instrument analysis. Chemometrics (PCA and OPLS-DA) was then used to analyze the differential chemical components. Furthermore, DPPH and ABTS assays were conducted to evaluate the antioxidant activities of *S. chinensis* and *S. sphenanthera*, and a gray relational analysis was employed to identify chemical components associated with antioxidant activity.

## 2. Results

### 2.1. Profiling of Non-Volatile Components

Samples of *S. chinensis* and *S. sphenanthera* were analyzed using UPLC-Q-TOF/MS. Given that most lignans from Schisandra show poor responses in negative ion mode, this study utilized positive ion mode for detection.

Identification was based on retention times, the primary and secondary mass spectrometry data of standards, and accurate molecular masses from a self-built database, which matched within an error range of δ = −5~5. Total ion chromatograms of *S. chinensis* and *S. sphenanthera* are shown in Figure S1, and component information is detailed in Table S1. The mean retention time of the schisandrol A peak (*m*/*z* 433.22) over five injections of a solution of sample N13 was 5.902 min, and the standard deviation was 0.008 min, with an RSD value of 0.141%. The mean retention time of the schisantherin A peak (*m*/*z* 537.21) over five injections of a solution of sample S8 was 9.498 min, and the standard deviation was 0.010 min, with an RSD value of 0.104%.

In total, 31 dibenzocyclooctadiene lignans were identified in 15 batches of *S. chinensis* ethanol extracts from different regions, and 40 dibenzocyclooctadiene lignans were identified in 15 batches of *S. sphenanthera* ethanol extracts, including 5 tetrahydrofuran lignans, 2 diarylbutane lignans, and 2 caryophyllene-type triterpenoids. Peaks 8 (gomisin T), 9 (zuihonin C), 22 (benzoylgomisin Q), 30 (schisphenins G), and 37 (benzoylgomisin U) were identified in *S. sphenanthera* ethanol extracts for the first time.

The comparative analysis in Figure S1 reveals that *S. chinensis* had a higher relative abundance of compound ion peaks between 6 and 9 min, while *S. sphenanthera* showed a higher relative intensity of compound ion peaks between 9 and 12 min. Compositionally, *S. sphenanthera* had a more diverse array of lignans, including diarylbutane, tetrahydrofuran, and dibenzocyclooctadiene types. Twelve common components were found in both *S. chinensis* and *S. sphenanthera*. Notably, peak 10 (schisandrol A) was significantly higher in *S. chinensis*, whereas peaks 30 (schisantherin C), 31 (schisantherin A), 32 (schisantherin B), and 44 (schisandrin A) were significantly higher in *S. sphenanthera*.

### 2.2. Profiling of Volatile Components

Total ion chromatograms of volatile oils from *S. chinensis* and *S. sphenanthera* obtained via GC-MS are presented in Figure S2. The peak times were concentrated between 5 and 7.5 min, 15 and 30 min, and 47.5 and 52.5 min. In total, 37 components were identified in the volatile oil of *S. chinensis*, including 8 monoterpenes, 20 sesquiterpenes, and 9 aliphatic compounds, such as olefins, acetylenes, alcohols, ketones, fatty acids, and their esters. Ylangene was the most abundant component in *S. chinensis*, consistent with previous reports. The mean retention time of the ylangene peak over five injections of volatile oil from sample N13 was 17.468 min, and the standard deviation was 0.067 min, with an RSD value of 0.384%. Tricyclo[2.2.1.0(26)]heptane 17-dimethyl-7-(4-methyl-3-pentenyl)-(−) was the most abundant component in *S. sphenanthera*, which was also consistent with previous reports. The mean retention time of the peak mentioned above over five injections of volatile oil from sample S8 was 20.722 min, and the standard deviation was 0.014 min, with an RSD value of 0.068%. Detailed information related to the volatile oil components of *S. chinensis* and *S. sphenanthera* is shown in Table 1. Nine common components were revealed in the GC-MS spectra of *S. chinensis* and *S. sphenanthera*: α-pinene, β-pinene, ylangene, bicyclo[5.2.0]nonane-2-methylene-488-trimethyl-4-vinyl, α-amorphene, γ-muurolene, trans-α-bergamotene, trans-nerolidol, and cubenol. This indicates that the compositions of the volatile oils were largely consistent, but there were substantial differences in the specific components between the two species.

### 2.3. PCA Results

**Figure 1:**
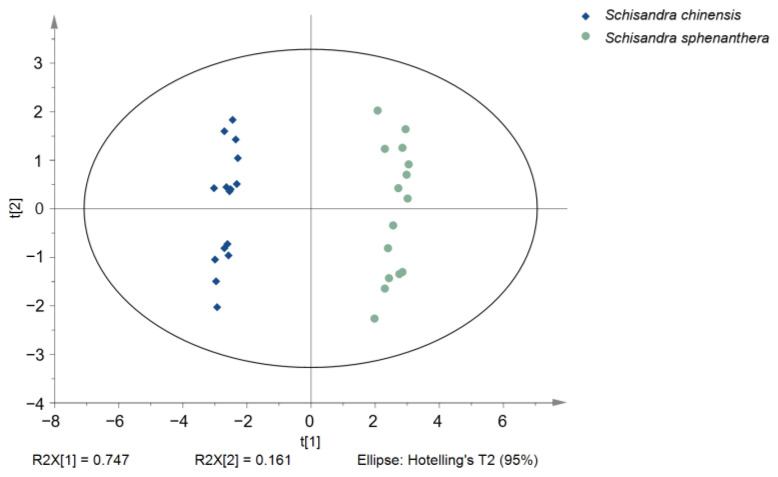
PCA analysis of *S. chinensis* and *S. sphenanthera*.

PCA is a mathematical statistical tool for categorizing relatively homogeneous groups in an unsupervised manner [13]. UPLC-MS data and GC-MS data from 15 batches of *S. chinensis* and 15 batches of *S. sphenanthera* were fused via low-level fusion into a large dataset, resulting in a 30 × 21 matrix. As shown in the score plot (Figure 1), 30 samples of *S. chinensis* and *S. sphenanthera* were separated into two groups along the t[1] axis, with *S. chinensis* on the left and *S. sphenanthera* on the right, indicating significant chemical differences between them. The first principal component contributed 74.7%, and the second principal component contributed 16.1%, with a cumulative explanation rate of 90.7% for the two principal components.

### 2.4. OPLS-DA Results

**Figure 2:**
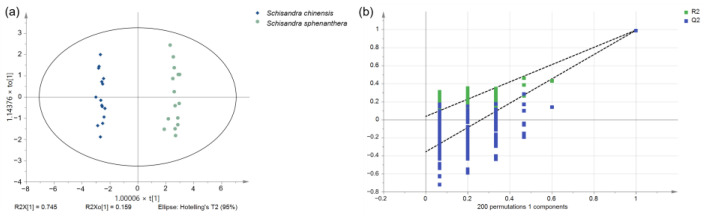

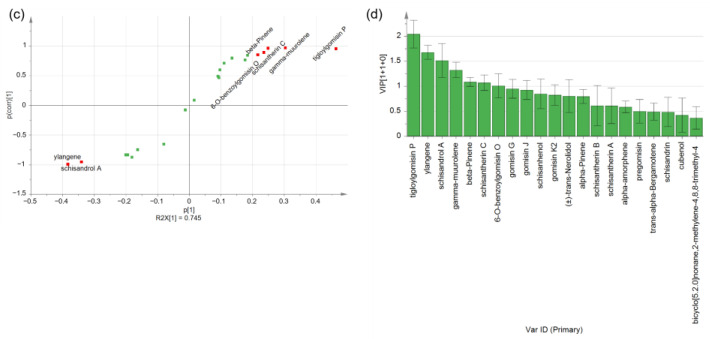
OPLS-DA analysis of *S. chinensis* and *S. sphenanthera*. (**a**) Score plot; (**b**) loading plot; (**c**) VIP diagram; and (**d**) permutation test diagram.

OPLS-DA was further used to determine the differential chemical components of *S. chinensis* and *S. sphenanthera*. The screening criteria were |P(corr)[1]| > 0.5 and a VIP value > 1. R2 represents the model’s fitting effect, and Q2 represents the model’s predictive ability. The parameters resulting from model fitting were R2X = 0.903, R2Y = 0.991, and Q2(cum) = 0.989, indicating an accurate model. The differential chemical markers distinguishing *S. chinensis* and *S. sphenanthera* were tigloygomisin P, ylangene, schisandrol A, γ-muurolene, β-pinene, schisantherin C, and 6-O-benzyolgomisin O. In total, 200 permutation tests were conducted to evaluate whether the model was valid. The criteria for validity were as follows: the intercept of the Q2 regression line should be at or below zero, and all green R2 values to the left should be lower than the original point to the right. The result (Figure 2) showed that when the regression line of R2 and Q2 intersected the left vertical axis, the values of R2 and Q2 were 0.0294 and −0.363, respectively. This meant that the model was effective and reliable.

### 2.5. Heatmap Analysis of Differential Chemical Components

**Figure 3:**
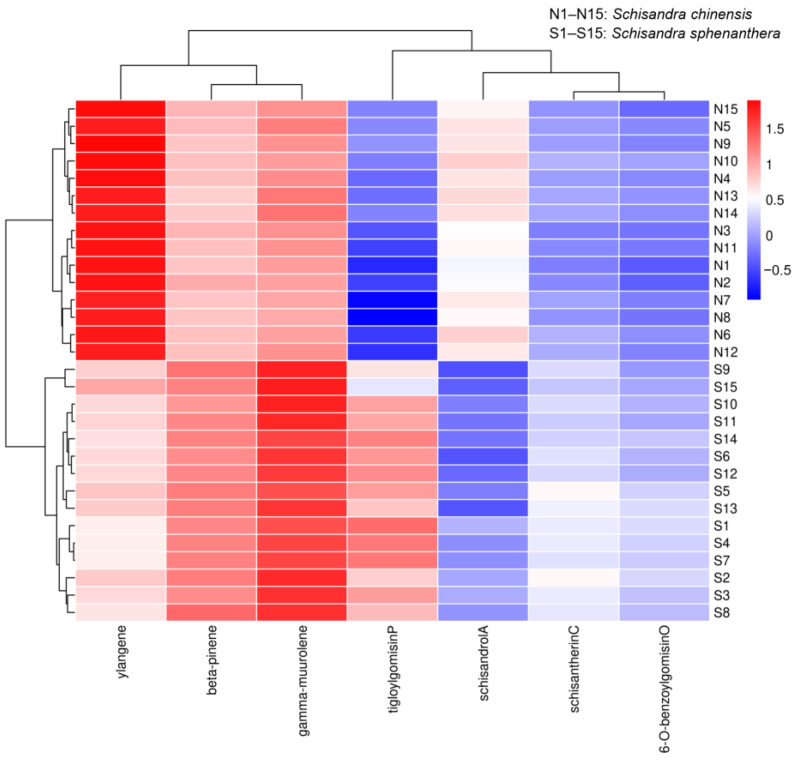
Heatmap analysis of differential chemical components.

As depicted in Figure 3, the non-volatile component schisandrol A and the volatile component ylangene had higher contents in *S. chinensis*. Meanwhile, the non-volatile components schisantherin C, tigloygomisin P, and 6-O-benzoylgomisin O and the volatile components β-pinene and γ-muurolene had higher contents in *S. sphenanthera*.

### 2.6. DPPH Assay Results

**Figure 4:**
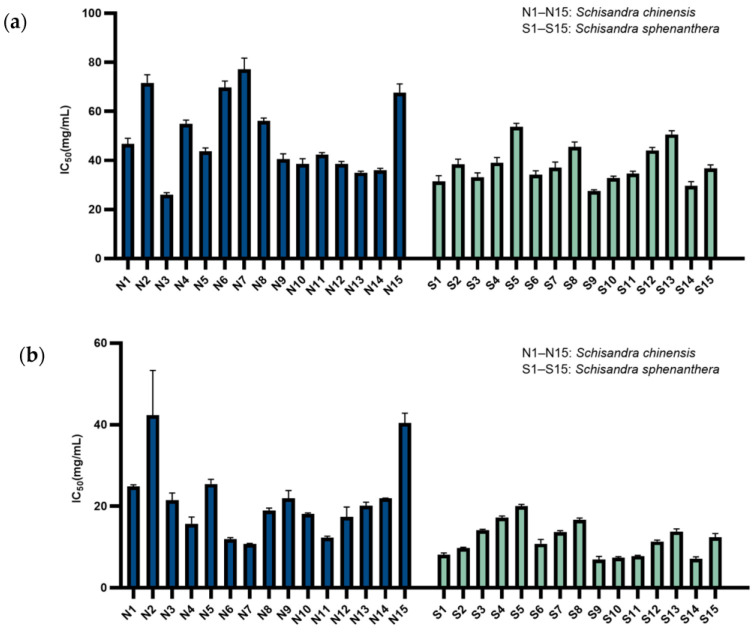
Antioxidant activity evaluated by (**a**) DPPH assays and (**b**) ABTS assays (x ± SD, *n* = 3).

The IC_50_ values for DPPH· scavenging by the ethanol extracts of 15 batches of *S. chinensis* and 15 batches of *S. sphenanthera* are shown in Figure 4a. The IC_50_ of the *S. chinensis* extract was 49.67 ± 15.63 μg/mL, while that of the *S. sphenanthera* extract was 37.94 ± 7.57 μg/mL. Overall, both the *S. sphenanthera* and *S. chinensis* extracts demonstrated comparable DPPH·-scavenging abilities, with no statistically significant differences observed between the two.

### 2.7. ABTS Assay Results

The IC_50_ values for ABTS·+ scavenging by the ethanol extracts of 15 batches of *S. chinensis* and 15 batches of *S. sphenanthera* are shown in Figure 4b. The IC_50_ of the *S. chinensis* extract for ABTS·+ scavenging was 37.94 ± 7.57 μg/mL, whereas that of the *S. sphenanthera* extract was 11.83 ± 4.09 μg/mL. The *S. sphenanthera* extract showed a superior ABTS·+-scavenging capacity compared to the *S. chinensis* extract, and the results were statistically significant (*p* = 0.003). Both *S. chinensis* and *S. sphenanthera* exhibited a stronger ability to scavenge ABTS·+ compared to DPPH, suggesting that the chemical bases for scavenging different radicals may involve different components or combinations of multiple components.

### 2.8. Gray Relational Analysis

There was a consistent material basis for the DPPH·− and ABTS·+-scavenging activities of *S. chinensis* and *S. sphenanthera*. In *S. chinensis*, the correlations between the components and the antioxidant activity ranged from 0.708 to 0.940. Schisandrol A, gomisin G, schisantherin C, pregomisin, gomisin J, and schisantherin B ranked among the top 10 correlations for both the DPPH·− and ABTS·+-scavenging activities, identifying these components as the primary contributors to the antioxidant activity of *S. chinensis*. In *S. sphenanthera*, the correlations between the components and the antioxidant activity ranged from 0.834 to 0.978. Gomisin K2, schisantherin B, gomisin J, pregomisin, schisantherin C, schisandrin, gomisin G, schisantherin A, schisanhenol, and α-pinene were the top 10 components, which identified them as the primary contributors to the antioxidant activity of *S. sphenanthera*. Studies have shown that lignans with phenolic hydroxyl groups exhibit significantly higher radical-scavenging capacities than those without these functional groups. Schisanhenol contains one hydroxyl group, and gomisin J contains two hydroxyl groups, making their antioxidant effects significantly stronger than those of other lignans [14,15] This supports the reliability of the results of the gray relational analysis. Combining findings from the differential chemical components, gray relational analysis, and heatmap analysis, schisantherin C was identified as a marker for differentiating *S. chinensis* and *S. sphenanthera*, as well as for characterizing antioxidant activity.

## 3. Discussion

This study utilized UPLC-QTOF/MS and GC-MS to conduct a comprehensive comparative analysis of the chemical profiles and antioxidant activities of *S. chinensis* and *S. sphenanthera*. The results revealed significant disparities in the compositions of both volatile and non-volatile components between the two species, which are crucial for their identification and effective use as medications.

In total, 31 non-volatile components and 37 volatile components were identified in *S. chinensis*, while *S. sphenanthera* contained 40 non-volatile components and 34 volatile components. Notably, *S. sphenanthera* exhibited more diverse non-volatile components, which may have contributed to its stronger antioxidant activity. Key differential markers were identified for the two species. For the non-volatile components, tigloylgomisin P, schisandrol A, schisantherin C, and 6-O-benzoylgomisin O were significant for distinguishing *S. chinensis* from *S. sphenanthera*. The differential non-volatiles schisandrol A, tigloylgomisin P, and 6-O-benzoylgomisin O were in accordance with a study by Li et al. [16]. Volatile components such as ylangene, γ-muurolene, and β-pinene also served as essential differentiators. The differential volatile ylangene was also observed in studies by Fu et al. [17], Guo et al. [8], and Shen et al. [18]. These markers not only help distinguish the two species but also provide insights into their potential therapeutic applications.

The antioxidant activities were evaluated using DPPH and ABTS assays. The results indicated that the ethanolic extracts of *S. sphenanthera* exhibited stronger antioxidant activity compared to those of *S. chinensis*. For *S. chinensis* and *S. sphenanthera,* the IC_50_ values for DPPH· scavenging were 49.67 ± 15.63 μg/mL and 37.94 ± 7.57 μg/mL, while for ABTS·+ scavenging they were 37.94 ± 7.57 μg/mL and 11.83 ± 4.09 μg/mL, respectively.

Gray relational analysis further identified the key components contributing to the antioxidant activity. In *S. chinensis*, schisandrol A, gomisin G, schisantherin C, pregomisin, gomisin J, and schisantherin B were closely associated with its antioxidant activity. According to the results of Pi et al. [12], the content of schisandrol A is positively correlated with the antioxidant activity of *S. chinensis*. For *S. sphenanthera*, gomisin K2, schisantherin B, gomisin J, pregomisin, schisantherin C, schisandrin, gomisin G, schisantherin A, schisanhenol, and α-pinene were the primary contributors. These findings align with previous studies indicating that lignans with phenolic hydroxyl groups have significantly stronger radical-scavenging capacities [15]. The identification of differential chemical markers and the evaluation of antioxidant activities provide a foundation for further research into the therapeutic applications of these species. This can be particularly beneficial when developing functional foods and nutraceuticals aimed at mitigating oxidative stress-related diseases [19].

This study systematically compared the chemical compositions and antioxidant activities of *Schisandra chinensis* and *Schisandra sphenanthera* using UPLC-MS and GC-MS techniques. Compared to previous research, significant advancements in both compound identification and functional analysis were noted. A study by Zhang et al. [2] provided a comprehensive review of the chemical constituents and hepatoprotective effects of *Schisandra sphenanthera* and *Schisandra chinensis*, identifying a range of active compounds such as lignans, essential oils, flavonoids, and triterpenes. Building upon this foundation, our study complemented these findings by offering a detailed comparison of the chemical profiles of these two species. Using UPLC-MS and GC-MS techniques, we identified 31 non-volatile compounds and 37 volatile compounds in *S. chinensis*, as well as 40 non-volatile compounds and 34 volatile compounds in *S. sphenanthera*. Additionally, our research further explored their antioxidant activities, providing valuable insights into their functional properties. Together, these studies contributed to a more comprehensive understanding of the chemical compositions and therapeutic potentials of these two species. A study by Guo et al. [8] differentiated the metabolite profiles of *S. chinensis* and *S. sphenanthera* using UPLC-MS and GC-MS. It primarily focused on identifying key differential metabolites such as lignans and volatile compounds. While the current study also confirmed these differential compounds, additional functional differences in antioxidant activity were analyzed. Guo et al.’s study emphasized metabolite profiling, whereas this research added an additional layer by revealing the greater antioxidant potential of *S. sphenanthera*. Wang et al. [15] evaluated the antioxidant activity of *S. chinensis* using lipid peroxidation inhibition assays (LPIL, LPIK, and LPIB) and DPPH radical-scavenging assays. In contrast, this study focused on the antioxidant activities of both *S. chinensis* and *S. sphenanthera* through DPPH and ABTS assays. While lipid peroxidation assays were not included, both studies consistently demonstrated that schisandrin and schisandrol B were significant contributors to antioxidant activity. Additionally, Pi et al. [12] reported a negative correlation between deoxyschizandrin and antioxidant activity, but deoxyschizandrin was not detected as a significant compound in this study. In this regard, the results are consistent, as deoxyschizandrin was not identified as a major antioxidant compound in either study. In conclusion, this study not only confirmed key findings from previous research but also provided additional insights by revealing *S. sphenanthera*’s greater antioxidant potential and expanding upon the functional roles of these compounds in antioxidant mechanisms.

This study expanded on previous work that utilized LC-MS- and GC-MS-based metabolomic approaches to differentiate *Schisandra* species. While past studies identified a limited set of marker compounds, this study significantly expanded the chemical profiling, identifying 31 non-volatile compounds and 37 volatile compounds in *S. chinensis*, as well as 40 non-volatile compounds and 34 volatile compounds in *S. sphenanthera*. Additionally, for the first time, antioxidant activity was evaluated, revealing *S. sphenanthera*’s stronger ability to scavenge ABTS radicals. Through innovative data fusion methods like PCA and OPLS-DA, new differential markers such as tigloygomisin P, schisandrol A, and β-pinene were identified. Furthermore, this study provided insight into the distinct antioxidant mechanisms of these two species, highlighting differences in the compounds contributing to their DPPH- and ABTS-scavenging activities. These findings contribute valuable knowledge for the quality control and therapeutic application of these species.

Moreover, this study underscores the importance of comprehensive chemical profiling in quality control and standardization of herbal medicines. The identified markers can be used to develop more precise and reliable methods for the authentication and quality assessment of *S. chinensis* and *S. sphenanthera*. Future research should further explore the spectrum–effect relationships by employing various data processing methods, such as principal component analysis and regression analysis, in order to establish more robust links between chemical composition and biological activities. Additionally, an investigation into alternative pharmacological effects, such as hepatoprotection and treatment of bronchial asthma, will contribute to a more comprehensive understanding of the efficacy differences between these two species.

## 4. Materials and Methods

### 4.1. Plant Materials

From December 2019 to February 2023, 15 batches of *S. chinensis* were collected from the Lotus Pond Chinese herbal medicine market in Sichuan, the Bozhou Chinese herbal medicine market in Anhui, and the Anguo Chinese herbal medicine market in Hebei, China. They included 3 batches from Heilongjiang (designated as N1–N3), 5 batches from Liaoning (designated as N4–N8), 4 batches from Jilin (designated as N9–N12), 3 batches from Inner Mongolia (designated as N13–N14), and 1 batch (designated as N15) from Shandong. In addition, 15 batches of *S. sphenanthera* were collected, including 8 batches from Shaanxi (designated as S1–S8), 3 batches from Shanxi (designated as S9–S11), 2 batches from Henan (designated as S12–S13), 1 batch from Yunnan (designated as S14), and 1 batch from Anhui (designated as S15).

### 4.2. Chemicals and Reagents

Reference compounds, schisandrol B, schisandrin A, schisandrin B, schisandrol C, schisantherin A, schisantherin B, schisantherin C, schisanhenol, gomisin D, gomisin G, angeloylgomisin H, gomisin J, gomisin L1, gomisin M2, and gomisin N were purchased from Must Bio-Technology Co., Ltd. (Chengdu, China). Schisandrol A and schisantherin E were purchased from NatureStandard Standard Technology Co., Ltd. (Shanghai, China). Anwulignan was purchased from Meisai Biotechnology Co., Ltd. (Chongqing, China). The purities of all reference compounds were greater than 98%. 1,1-Diphenyl-2-picrylhydrazyl free radicals were purchased from MedChemExpress LLC (Shanghai, China). 3-Ethylbenzthiazoline-6- sulphonate was purchased from Macklin Biochemical Technology Co., Ltd. (Shanghai, China). MS- and HPLC-grade formic acid and methanol were bought from Fisher Scientific (Fair Lawn, NJ, USA). HPLC-grade acetonitrile was bought from Supelco Inc. (Bellefonte, PA, USA). Ultrapure water was obtained from Watsons (Guangzhou, China). Analytical-grade methanol, ethanol, and other reagents were purchased from Sinopharm Chemical Reagent Co., Ltd. (Shanghai, China).

### 4.3. UPLC-Q-TOF/MS Analysis

UPLC-Q-TOF/MS analysis, following methods from previous publications by our research group [20], then prepared the sample solution. To ensure accurate mass measurement throughout the analytical run, leucine-enkephalin was injected as an external standard.

### 4.4. GC-MS Analysis

In our preliminary work, we conducted a comparison of drying processes and found that vacuum drying required the shortest duration. Moreover, the main components after drying were largely consistent with those reported by Sheng et al. [21]. Therefore, we chose the vacuum drying method. Powders of *S. chinensis* and *S. sphenantherae* (20 g) were immersed in 300 mL of distilled water in a 500 mL round-bottom flask and extracted for 6 h in an essential oil extractor. During the extraction process, the solution in the flask was kept slightly boiling. As reported by Shen et al. [18], 2 mL of ethyl acetate was used as a receptor solvent in the essential oil extractor. After cooling to ambient temperature, the ethyl acetate layer was collected and dehydrated using anhydrous sodium sulfate. Then, it was diluted 4 times. The dilution was analyzed via GC-MS after filtering through a 0.22 μm membrane.

The volatile composition of ethyl acetate was analyzed via GC-MS QP2010(Shimadzu, Tokyo, Japan). Volatile compounds were separated using a WM-5MS capillary column (30 m × 0.25 mm i.d., 0.25 μm film thickness) (Welch, Shanghai, China). The carrier gas was helium (purity > 99.99%). The injection temperature was 240 °C, the split ratio was 20:1, and the injection volume was 1 μL. A temperature-increase program was used as follows: The initial temperature was 70 °C, which was held for 2 min. The temperature was then increased to 120 °C at a rate of 8 °C/min, held for 15 min, increased to 140 °C at a rate of 2 °C/min, held for 10 min, increased to 165 °C at a rate of 5 °C/min, increased to 240 °C at a rate of 8 °C/min, and held for 2 min. Mass scan spectra were recorded from 35 to 500 amu.

### 4.5. DPPH Assay

A DPPH assay was conducted according to the method described by Liu et al. with a few modifications [22]. First, 10 μL of a sample solution was drawn and added to 190 μL of a 0.05 mg/mL DPPH solution. They were mixed evenly as an experimental group. In addition, 10 μL of a sample solution was added to 190 μL of methanol as a control, while 10 μL of an ethanol solution was added to 190 μL of a DPPH solution as a zero-regulating group. The samples reacted away from light for 30 min at room temperature. Absorbance was measured at a wavelength of 517 nm. Each sample was run in triplicate and averaged. Scavenging activity (%) = [1 − (Aexperimental − control)/(Azero − regulating)] × 100%. The dose–effect curve was plotted with the clearance rate as the ordinate and the concentration of the sample solution as the abscissa. The concentration of the sample at a clearance rate of 50% (IC_50_, μmol/L) was calculated based on the dose–effect curve.

### 4.6. ABTS Assay

An ABTS assay was carried out using a modified version of the method described by Nijat et al. [23]. An ABTS·+ deionized water solution (1.92 mg/mL) was mixed with K2S2O8 (2.45 mM) and kept in a dark place for 24 h at room temperature. The configured ABTS·+ solution was diluted with water to regulate the absorbance at 734 nm, which was 0.70 ± 0.05. Then, 10 μL of a sample solution and 190 μL of an ABTS·+ solution were mixed as an experimental group. In addition, 10 μL of a sample solution was added to 190 μL of methanol as a control, while 10 μL of water was added to 190 μL of an ABTS·+ solution as a zero-regulating group. All samples reacted in the dark for 6 min at room temperature, and absorbance was measured at 734 nm. Each sample was run in triplicate and averaged. Scavenging activity (%) = [1 − (Aexperimental − Acontrol)/(Azero − regulating)] × 100%. The dose–effect curve was plotted with the clearance rate as the ordinate and the concentration of the sample solution as the abscissa. The concentration of the sample at a clearance rate of 50% (IC_50_, μmol/L) was calculated based on the dose–effect curve.

### 4.7. Data Processing

MassLynx4.1 software and the UNIFI platform were employed to identify non-volatile compounds. The non-volatile compounds in *S. chinensis* and *S. sphenanthera* were identified by matching the MS data of a reference standard, referring to the retention behavior and relative contents of the compound in the literature [24,25,26,27,28]. The strongest signals from the extracted ion chromatograms of the corresponding molecular peak obtained via UPLC-Q-TOF/MS and GC-MS were used to determine the reproducibility of the total retention times. A compound database was constructed manually. The collected information contained compound names, molecular formulas, cleavage patterns, and chemical structure files. MSe continuum data and the self-established component database were imported into the UNIFI platform to assist the process of compound identification. The selected adduct ions included [M + H]^+^, [M + NH4]^+^, [M + Na]^+^, [M + K]^+^, [2M + H]^+^, [2M + NH4]^+^, [2M + Na]^+^, and [2M + K]^+^. Volatile compounds were identified by referencing spectral data from the NIST 17 library. The identification criteria were that the similarity was greater than 70 and the peak relative abundance was greater than 0.1%. Relative abundance (%) was calculated by dividing the area of each peak by the sum of all detected peak areas multiplied by 100 [13].

All raw UPLC-Q-TOF/MS data (raw format) were imported into Progenesis QI 2.1 (Waters, Milford, MA, USA) for peak alignment, peak picking, and normalization. All GC-MS data (.text format) were processed based on “Similarity analyses of chromatographic herbal fingerprints” for peak alignment. Then, tables containing the peak retention times, peak areas, and mass-to-charge ratios (*m/z*) in the GC-MS and UPLC MS data were output. The output tables were manipulated in Excel (Microsoft Office 2017) to produce a large dataset with a “low-level” data fusion approach. The peak area detected in each sample was normalized (area of the peak/sum of the areas of all peaks in the sample × 10,000) [29]. The normalized datasets were imported into the SIMCA P+ statistical software for multivariate analyses. With Pareto scaling, principal component analysis (PCA) and orthogonal partial least squares discriminant analysis (OPLS-DA) were performed. PCA was performed to observe the categorical trends in *S. chinensis* and *S. sphenanthera*. An OPLS-DA model was used to obtain the differential chemical markers of *S. chinensis* and *S. sphenanthera*. In the discriminant model, an S-plot was used to visualize the differential compound with the highest correlation and covariance.

### 4.8. Statistical Analysis

The Mann–Whitney U test and t-test were performed using IBM SPSS Statistics (IBM, Armonk, NY, USA), and variable influence on projection (VIP) values were used to screen the differential chemical markers in combination with the *p*-values from the Mann–Whitney U test. All determinations of antioxidant activity via the DPPH and ABTS assays were carried out in triplicate. Experimental data were expressed as means ± SDs. Factors and variables were entered into the SPSSAU online platform to conduct a gray correlation analysis.

## 5. Conclusions

This study successfully established the chemical profiles of *Schisandra chinensis* and *Schisandra sphenanthera* and assessed their antioxidant activities. The results revealed significant differences between the two species, with *S. sphenanthera* demonstrating a stronger ABTS radical-scavenging ability compared to *S. chinensis*. Key differential compounds were identified, including tigloygomisin P, ylangene, schisandrol A, γ-muurolene, β-pinene, schisantherin C, and 6-O-benzyolgomisin O. These identified markers, along with the key antioxidant components, provide valuable insights into the distinct therapeutic potentials of both species. Furthermore, these findings contribute to the standardization and quality control of these medicinal plants, supporting their safe and effective use in both traditional and modern medicinal practices.

## Figures and Tables

**Table 1:** Volatile components of *S. chinensis* and *S. sphenanthera*.

Peak No.	Retention Time/min	Compound	Formula	Plant Source		Classification
				*S. chinensis*	*S. sphenanthera*	
1	5.106	α-Pinene	C_10_H_16_	+	+	monoterpene
2	5.377	Camphene	C_10_H_16_	+	−	monoterpene
3	6.025	β-Pinene	C_10_H_16_	+	+	monoterpene
4	6.552	Bornylene	C_10_H_16_	+	−	monoterpene
5	6.695	m-Cymene	C_10_H_14_	+	−	monoterpene
6	6.78	D-Limonene	C_10_H_16_	+	−	monoterpene
7	7.33	γ-Terpinene	C_10_H_16_	+	−	monoterpene
8	9.808	4-Terpineol	C_10_H_18_O	+	−	alcohol
9	11.246	2-Isopropyl-5-methylanisole	C_11_H_16_O	+	−	ether
10	13.072	Bornyl acetate	C_12_H_20_O_2_	+	−	ester
11	15.363	3-Carene	C_10_H_16_	−	+	monoterpene
12	16.118	α-Cubebene	C_15_H_24_	−	+	sesquiterpene
13	16.893	α-Bisabolene	C_15_H_24_	−	+	sesquiterpene
14	17.127	1,2,4-Methenoazulene	C_15_H_24_	+	−	sesquiterpene
15	17.432	Ylangene	C_15_H_24_	+	+	sesquiterpene
16	17.687	Copaene	C_15_H_24_	−	+	sesquiterpene
17	18.248	Cyclobuta[1,2:3,4]dicyclopentene,decahydro-3a-methyl-6-methylene-1-(1-methylethyl)-, (1S,3aS,3bR,6aS,6bR)	C_15_H_24_	−	+	sesquiterpene
18	19.37	Naphthalene,1,2,3,4,4a,5,6,8a-octahydro-7-methyl-4-methylene-1-(1-methylethyl)-,(1α,4aβ,8aα)	C_15_H_24_	−	+	sesquiterpene
19	20.475	Sativene	C_15_H_24_	+	−	sesquiterpene
20	20.72	Tricyclo[2.2.1.0(2,6)]heptane,1,7-dimethyl-7-(4-methyl-3-pentenyl)	C_15_H_24_	−	+	sesquiterpene
21	21.091	Isoledene	C_15_H_24_	−	+	sesquiterpene
22	21.356	(−)-Thujopsene	C_15_H_24_	+	−	sesquiterpene
23	23.561	β-(Z)-Farnesene	C_15_H_24_	+	−	sesquiterpene
24	23.945	Germacrene D	C_15_H_24_	−	+	sesquiterpene
25	24.137	α-cis-Bisabolene	C_15_H_24_	+	−	sesquiterpene
26	24.374	α-Acoradiene	C_15_H_24_	+	−	sesquiterpene
27	25.145	Cyclohexene,4-ethenyl-4-methyl-3-(1-methylethenyl)	C_15_H_24_	−	+	sesquiterpene
28	25.258	Bicyclo[5.2.0]nonane, 2-methylene-4,8,8-trimethyl-4-viny	C_15_H_24_	+	+	sesquiterpene
29	25.546	α-Amorphene	C_15_H_24_	+	+	sesquiterpene
30	26.007	α-Selinene	C_15_H_24_	+	−	sesquiterpene
31	26.676	Naphthalene,1,2,4a,5,8,8a-hexahydro-4,7-dimethyl-1-(1-methylethyl)	C_15_H_24_	+	−	sesquiterpene
32	27.185	β-Himachalene	C_15_H_24_	+	−	sesquiterpene
33	27.413	α-Longipinene	C_15_H_24_	−	+	sesquiterpene
34	27.79	Cadina-1(10)	C_15_H_24_	+	−	sesquiterpene
35	27.798	Benzene,1-methyl-4-(1,2,2-trimethylcyclopentyl)-, (R)	C_15_H_22_	−	+	alkene
36	27.993	β-Bisabolene	C_15_H_24_	+	−	sesquiterpene
37	28.308	γ-Muurolene	C_15_H_24_	+	+	sesquiterpene
38	29.172	Cedrene	C_15_H_24_	+	−	sesquiterpene
39	29.298	Naphthalene,1,2,4a,5,8,8a-hexahydro-4,7-dimethyl-1-(1-methylethyl)-,(1S,4aR,8aS)	C_15_H_24_	−	+	sesquiterpene
40	29.715	trans-α-Bergamotene	C_15_H_24_	+	+	sesquiterpene
41	30.312	α-Muurolene	C_15_H_24_	−	+	sesquiterpene
42	31.015	γ-Elemene	C_15_H_24_	−	+	sesquiterpene
43	31.476	Alloaromadendrene	C_15_H_24_	+	−	sesquiterpene
44	31.579	β-Vatirenene	C_15_H_22_	−	+	alkene
45	32.353	(±)-trans-Nerolidol	C_15_H_26_O	+	+	alcohol
46	33.19	(−)-Spathulenol	C_15_H_24_O	−	+	alcohol
47	33.466	Caryophyllene oxide	C_15_H_24_O	−	+	ether
48	35.812	Aromadendreneoxide-(1)	C_15_H_24_O	−	+	ether
49	36.761	Alloaromadendreneoxide-(2)	C_15_H_24_O	−	+	ether
50	37.408	Cubenol	C_15_H_26_O	+	+	alcohol
51	38.555	trans-(Z)-α-Bergamotol	C_15_H_24_O	−	+	alcohol
52	39.485	α-Santalol	C_15_H_24_O	−	+	alcohol
53	39.829	Ledol	C_15_H_26_O	−	+	alcohol
54	41.157	1H-cycloprop[e]azulen-7-ol, decahydro-1,1,7-trimethyl-4-methylene-,[1ar-(1aα,4aα,7β,7aβ,7bα)]	C_15_H_24_O	−	+	alcohol
55	42.162	α-Santalol	C_15_H_24_O	−	+	alcohol
56	47.815	Cis-cyclohexane, 1, 2-diethenyl-4-(1-methylethylidene)	C_13_H_20_	+	−	alkene
57	48.338	Docosahexaenoic acid methyl ester	C_14_H_2_O	+	−	ester
58	48.807	2,4a-Methanonaphthalen-7(4aH)-one,1,2,3, 4,5,6-hexahydro-1,1,5,5-tetramethyl-,(2s-cis)	C_15_H_22_O	+	−	alkone
59	49.506	Bicyclo[7.2.0]undec-4-ene,4,11,11-trimethyl-8-methylene	C_15_H_24_	+	−	sesquiterpene
60	49.968	2,5,6-Trimethylhepta-1,3,6-triene	C_10_H_16_	+	−	monoterpene
61	50.315	Isomer of Bicyclo[5.2.0]nonane, 2-methylene-4,8,8-trimethyl-4-vinyl	C_15_H_24_	+	−	sesquiterpene
62	51.099	Farnesyl acetate	C_17_H_28_O_2_	+	−	ester

“+” indicates the presence of the compound, and “−” indicates the absence of the compound.

## Data Availability

Data will be made available on request.

## Supplementary Materials

The following supporting information can be downloaded at https://www.mdpi.com/article/10.3390/molecules29204865/s1, Table S1: Retention time and mass spectrometry information of the non-volatile components of *S. chinensis* and *S. sphenanthera*. Figure S1. The total ion chromatogram from UPLC-Q-TOF/MS. Figure S2. The total ion chromatogram from GC-MS. Figure S3. The key antioxidant compounds in *S. sphenanthera*. Figure S4. The key antioxidant compounds in *S. chinensis*.

## References

[1] Committee C.P. (2020). Pharmacopoeia of the People’s Republic of China.

[2] Zhang M.X., Huang G.Y., Bai Y.Q., Li H., Yang B. (2021). Advances in chemical constituents and hepatoprotective effect of *Schisandrae sphenantherae* fructus and *Schisandrae chinensis* fructus. China J. Chin. Mater. Med..

[3] Qiu H.T., Zhao X.P., Li Z., Wang L.L., Wang Y. (2015). Study on main pharmacodynamic effects for schisandra lignans based upon network pharmacology. China J. Chin. Mater. Med..

[4] Jeong H.J., Han N.R., Kim K.Y., Choi I.S., Kim H.M. (2014). Gomisin A decreases the LPS-induced expression of iNOS and COX-2 and activation of RIP2/NF-κB in mouse peritoneal macrophages. Immunopharm Immunot..

[5] Ip S.P., Poon M.K., Wu S.S., Che C.T., Ng K.H., Kong Y.C., Ko K.M. (1995). Effect of schisandrin B on hepatic glutathione antioxidant system in mice: Protection against carbon tetrachloride toxicity. Planta Med..

[6] Fan X.M., Chen P., Jiang Y.M., Wang Y., Tan H.S., Zeng H., Wang Y.T., Qu A.J., Gonzalez F.J., Huang M. (2015). Therapeutic efficacy of Wuzhi tablet (*Schisandra sphenanthera* Extract) on acetaminophen-induced hepatotoxicity through a mechanism distinct from N-acetylcysteine. Drug Metab. Dispos..

[7] Han Z.Z., Yang Y., Jia H.M., Wei M., Ma Q., Zhan R.T., Chen W.W., Zhou Z.M. (2017). Chemical comparison of cultivated and wild type *Flos Chrysanthemi Indici* base on plant metabolomics and determination of the chemical constituents by UPLC. Chin. J. Pharm. Anal..

[8] Guo Z.Y., Zhao A.H., Chen T.L., Xie G.X., Zhou M.M., Qiu M.F., Jia W. (2012). Differentiation of *Schisandra chinensis* and *Schisandra sphenanthera* using metabolite profiles based on UPLC-MS and GC-MS. Nat. Prod. Res..

[9] Zhang X.Y., Liu J.S., Gao S.M., Chen R.C., Qi Y.D., Zhang B.G., Liu H.T., Xiao P.G. (2019). Research methods and applications progress on spectrum-effect relationships in study of traditional Chinese medicine. China J. Chin. Mater. Med..

[10] Willcox J.K., Ash S.L., Catignani G.L. (2004). Antioxidants and prevention of chronic disease. Crit. Rev. Food Sci. Nutr..

[11] Davies K.J.A. (2019). Free radicals and redox regulation in ageing. Free Radic. Biol. Med..

[12] Pi Z.F., Hou G.Y., Ai J., Song F.R., Liu Z.Q., Liu S.Y. (2012). Correlation between lignan content and antioxidant activity in *Schisandra chinensis* studied by chemometric methods. China J. Chin. Mater. Med..

[13] Cannavacciuolo C., Pagliari S., Giustra C.M., Carabetta S., Guidi Nissim W., Russo M., Branduardi P., Labra M., Campone L. (2023). LC-MS and GC-MS data fusion metabolomics profiling coupled with multivariate analysis for the discrimination of different parts of Faustrime Fruit and evaluation of their antioxidant activity. Antioxidants.

[14] Lin T.J., Liu G.T., Li X.J., Zhao B.L., Xin W.J. (1989). Scavenging effect of *Schisandra chinensis* on reactive oxygen radicals. Chin. J. Pharmacol. Toxicol..

[15] Wang Z.M. (2016). Study on the Lignans Contents of Schisandrae Chinensis Fructus. Master’s Thesis.

[16] Li W., Liu Y.L., Song Y.G., Liu K.Y., Su D., Feng Y.L., Li Y. (2015). Rapid identification on chemical constituents in fruits of *Schisandra sphenanthera* and *Schisandra chinensis* and discrimination markers by UPLC-QTOF-MS combined with OPLS-DA. Chin. Herb. Med..

[17] Fu S.L., Chen B., Yao S.Z. (2009). Comparative analysis of chemical constituents in fruits of *Schisandra chinensis* (Turcz. )Baill. and *Schisandra sphenanthera* Rehd. et Wils. Chin. J. Pharm. Anal..

[18] Shen Z.Y., Chen X.H., Xiong L., Bi K.S. (2008). Establishment and analysis of GC fingerprints for evaluating the quality of *Schisandra chinensis* and *Schisandra sphenanthera*. J. Shenyang Pharm. Univ..

[19] Mocan A., Schafberg M., Crișan G., Rohn S. (2016). Determination of lignans and phenolic components of *Schisandra chinensis* (Turcz.) Baill. using HPLC-ESI-ToF-MS and HPLC-online TEAC: contribution of individual components to overall antioxidant activity and comparison with traditional antioxidant assays. J. Funct. Foods.

[20] Lin L.J., Zhang M.X., Li H., Lan X.M., Wei X.L., Guo C., Yang B. (2023). Identification of Q-markers for *Schisandrae sphenantherae* Fructus in treating drug-induced liver injury based on network pharmacology, fingerprint and quantitative analysis. China J. Chin. Mater. Med..

[21] Sheng Y.H., Wang R., Wang Y.K., Li M.M., Hunag X., Chen C.B. (2022). Analysis of volatile components in *Schisandra chinensis* by GC-MS. West Chin. J. Pharm. Sci..

[22] Liu Y.R., Guo M.J., Yang L.X., Qu Y., Pan X.L., Yang B., Li H. (2024). Comparative study on main components and antioxidant activity of Scutellaria Radix from different genuine producing areas. Nat. Prod. Res. Dev..

[23] Nijat D., Lu C.F., Lu J.J., Abdulla R., Hasan A., Aidarhan N., Aisa H.A. (2021). Spectrum-effect relationship between UPLC fingerprints and antidiabetic and antioxidant activities of *Rosa rugosa*. J. Chromatogr. B Anal. Technol. Biomed. Life Sci..

[24] Huang X., Song F.R., Liu Z.Q., Liu S.Y. (2008). Structural characterization and identification of dibenzocyclooctadiene lignans in Fructus Schisandrae using electrospray ionization ion trap multiple-stage tandem mass spectrometry and electrospray ionization fourier transform ion cyclotron resonance multiple-stage tandem mass spectrometry. Anal. Chim. Acta.

[25] Zhou Y., Huang S.X., Pu J.X., Li J.R., Ding L.S., Chen D.F., Sun H.D., Xu H.X. (2011). Ultra performance liquid chromatography coupled with quadrupole time-of-flight mass spectrometric procedure for qualitative and quantitative analyses of nortriterpenoids and lignans in the genus Schisandra. J. Pharm. Biomed. Anal..

[26] Liu H.T., Zhang J., Li X.B., Qi Y.D., Peng Y., Zhang B.G., Xiao P.G. (2012). Chemical analysis of twelve lignans in the fruit of *Schisandra sphenanthera* by HPLC-PAD-MS. Phytomedicine.

[27] Liu H.T., Lai H.W., Jia X.Y., Liu J.S., Zhang Z., Qi Y.D., Zhang J., Song J.B., Wu C.M., Zhang B.G. (2013). Comprehensive chemical analysis of *Schisandra chinensis* by HPLC-DAD-MS combined with chemometrics. Phytomedicine.

[28] Zhang J.P., Guo X.F., Yang Y.Y., Zhao Z.Y., Li B.L., Liu Y.Y., Qi Y.X. (2022). Simultaneous determination of fifteen components in purified lignans from *Schisandrae chinensis* fructus by UPLC. Chin. J. Pharm. Anal..

[29] Charve J., Chen C., Hegeman A.D., Reineccius G.A. (2011). Evaluation of instrumental methods for the untargeted analysis of chemical stimuli of orange juice flavour. Flavour Fragr. J..

