# Supplementary material for: Characterization of Differences in Chemical Profiles and Antioxidant Activities of Schisandra chinensis and Schisandra sphenanthera Based on Multi-Technique Data Fusion
Source: Molecules. 2024 Oct 14;29(20):4865. doi: 10.3390/molecules29204865 (PMC11510710; doi:10.3390/molecules29204865)
Supplement: Supplementary file 1 [file molecules-29-04865-s001.zip › molecules-3206281-Supplementary.pdf]

Supplementary Materia

Characterization of Differences in Chemical Profiles and Antioxidant Activities of *Schisandra chinensis* and *Schisandra sphenanthera* based on Multi-Technique Data Fusion

Lujie Lin †, Zhuqian Tang †, Huijuan Xie, Lixin Yang, Bin Yang *, Hua Li *

Institute of Chinese Materia Medica, China Academy of Chinese Medical Sciences, Beijing 100700, P.R. China

†These autors contributed equally to this work.

*Correspondence: ybinmm@126.com (B.Y.); hli1976@icmm.ac.cn (H.L.); Tel.: +86-10-64093058 (B.Y. & H.L.)

Table Captions:

Table S1. Retention time and mass spectrometry information of the non-volatile components of *S. chinensis* and *S. sphenanthera*

Table S1. Retention time and mass spectrometry information of the non-volatile components of *S. chinensis* and *S. sphenanthera*

Peak No.	tR/min	Formula	Experimental value (m/z)	Theoretical value（m/z）	Fragment ions	Compound	Error/ppm	Plant Source	
								S. chinensis	*S. sphenanthera*
1	4.91	$C_{20}H_{22}O_5$	343.1533[M+H]+	343.1545	219.1007，204.0768，189.0544，161.0594	Chicanine	3.50	-	+
2	5.03	$C_{27}H_{34}O_7$	471.2377[M+H]+	471.2383	437.2289，419.2268，327.6404	Schisanchinin B	1.27	+	-
3	5.09	$C_{20}H_{22}O_5$	343.1533[M+H]+	343.1545	219.1007，204.0768，189.0544，161.0569	Isomer of chicanine	3.50	-	+
4	5.15	$C_{22}H_{26}O_6$	387.1792[M+H]+	387.1807	345.1325，314.1127，299.0918，241.0877	Neglschisandrin E	3.87	-	+
5	5.24	$C_{22}H_{26}O_7$	403.1742[M+H]+	403.1757	343.1170，330.1110	Schisphenlignan G	3.72	-	+
6	5.43	$C_{23}H_{30}O_7$	457.1608[M+K]+	457.1629	401.1933，369.1674，323.1277	Gomisin S	4.59	-	+
7	5.43	$C_{24}H_{32}O_7$	455.2035[M+Na]+	455.2046	415.2073，401.1933，384.1935，369.1714，338.1500	Isomer of schisandrol A	0.03	+	-
8	5.63	$C_{23}H_{30}O_7$	457.1608[M+K]+	457.1629	401.1933，369.1674，323.1277	Gomisin T	4.59	-	+
9	5.72	$C_{21}H_{24}O_5$	357.1691[M+H]+	357.1702	233.1185，218.0936，203.0698，175.0743	Zuihonin C	3.08	-	+
10	5.91*	$C_{24}H_{32}O_7$	471.1794[M+K]+	471.1785	415.2115，400.1860，384.1935，369.1674，338.1462	Schisandrol A	-1.91	+	+
11	6.13*	$C_{30}H_{34}O_9$	577.1865[M+K]+	577.184	417.1896，399.1800，357.1304，353.1406，342.1452	Schisantherin E	-4.33	-	+
12	6.26	$C_{28}H_{34}O_{10}$	531.222[M+H]+	531.223	485.2159，401.1564，383.1477，352.1304	Gomisin D	0.96	+	-
13	6.38*	$C_{22}H_{28}O_6$	389.1975[M+H]+	389.1964	374.1758，357.1652，342.1490，287.0911，227.0685	Gomisin J	-2.83	+	+
14	6.49	$C_{23}H_{28}O_7$	455.1467[M+K]+	455.1472	399.1800，384.1534，368.1620，353.1368，337.1426	Schisandrol B	1.37	+	-
15	7.16	$C_{28}H_{36}O_8$	523.2326[M+Na]+	523.2308	483.2353，401.1933，386.1687，370.1743	Tigloylgomisin H	0.38	+	-

16	7.32	$C_{22}H_{30}O_6$	390.2030[M]+	390.2042	237.1465，327.0704，167.0697	Pregomisin	3.08	+	+
17	7.57	$C_{23}H_{28}O_7$	417.1897[M+H]+	417.1913	399.1800，368.1620，338.1500	Epigomisin O	3.84	-	+
18	7.65	$C_{28}H_{36}O_8$	539.2054[M+K]+	539.2047	483.2353，401.1933，386.1687，370.1743	Angeloylgomisin H	1.34	+	-
19	7.72	$C_{28}H_{34}O_9$	553.1837[M+K]+	553.184	385.1604，371.1470，340.1309，325.1089	Gomisin E	0.54	-	+
20	7.84	$C_{23}H_{28}O_7$	455.1463[M+K]+	455.1472	399.1800，384.1574，368.162	Isomer of schisandrol B	1.98	-	+
21	7.9	$C_{30}H_{34}O_8$	561.1895[M+K]+	561.1891	505.2214，401.1933，386.1727，370.1743	Benzoylgomisin H	0.00	+	-
22	7.94	$C_{31}H_{36}O_9$	591.1980[M+K]+	591.1996	431.2060，399.1800，356.1608	Benzoylgomisin Q	2.71	-	+
23	8.14	$C_{29}H_{38}O_9$	569.2136[M+K]+	569.2153	431.2060，413.1965，401.1892，356.1608	Angeloylgomisin Q	-0.36	+	-
24	8.15	$C_{30}H_{32}O_9$	575.1685[M+K]+	575.1684	415.1740，397.1677，371.1470，325.1053	Isomer of gomisin G	-0.17	-	+
25	8.38*	$C_{30}H_{32}O_9$	575.1685[M+K]+	575.1684	415.1740，397.1677，384.1534，371.1470，325.1053	Gomisin G	-0.17	+	+
26	8.52	$C_{28}H_{34}O_9$	553.1838[M+K]+	553.184	415.1740，371.1470，343.1145	Gomisin F	0.94	+	-
27	8.65	$C_{30}H_{40}O_5$	481.2947[M+H]+	481.2954	445.2770，427.2604，381.1084	longipedlactone B	1.45	-	+
28	8.8	$C_{23}H_{30}O_6$	403.2117[M+H]+	403.2121	388.1893，371.1825，340.1654，325.1458，302.1135	Gomisin K1	0.25	+	-
29	8.95	$C_{23}H_{30}O_6$	403.2117[M+H]+	403.2121	388.1893，371.1825，340.1654，325.1458，302.1135	Gomisin K2	0.00	+	+
30	9.11*	$C_{28}H_{34}O_9$	553.1838[M+K]+	553.184	415.1740，371.1470，356.1260，343.1145，325.1053	Schisantherin C	0.36	+	+
31	9.42*	$C_{30}H_{32}O_9$	575.1685[M+K]+	575.1684	415.1740，371.1470，356.1260，343.1145，325.1053	Schisantherin A	-0.17	+	+
32	9.61*	$C_{28}H_{34}O_9$	553.1838[M+K]+	553.184	415.1740，371.1470，356.1260，343.1145，325.1053	Schisantherin B	0.36	+	+
33	9.83	$C_{29}H_{28}O_9$	559.1367[M+K]+	559.137	399.1432，381.1284，355.1154	Schisantherin D	0.54	-	+

34	10.15*	$C_{23}H_{30}O_6$	403.2117[M+H]+	403.2121	388.1852, 371.1825, 340.1654, 325.1421, 302.1135	Schisanhenol	0.99	+	+
35	10.61	$C_{22}H_{26}O_6$	386.1727[M]+	386.1729	369.1674, 355.1578, 325.1385, 227.0685	Gomisin M1	1.40	+	-
36	11.31	$C_{28}H_{34}O_9$	515.2294[M+H]+	515.2281	415.1782, 385.1644, 343.1183	Tigloylgomisin P	-2.52	+	+
37	11.61	$C_{30}H_{34}O_8$	561.1891[M+K]+	561.1891	401.1933, 383.1477, 369.1714	Benzoylgomisin U	0.00	-	+
38	11.73	$C_{22}H_{26}O_6$	386.1727[M]+	386.1729	369.1674, 355.1539, 325.1421	Gomisin L1	0.11	+	-
39	11.91	$C_{25}H_{30}O_8$	497.1570[M+K]+	497.1578	399.1800, 368.1620, 353.1368, 337.1426, 330.1072	Isomer of kadsurin	1.61	-	+
40	12.27	$C_{29}H_{40}O_{12}$	581.2579[M+H]+	581.2598	383.1477, 352.1295	schisandroside B	3.27	-	+
41	12.28	$C_{22}H_{26}O_6$	387.1809[M]+	386.1729	369.1674, 355.1539, 325.1421	Gomisin L2	-0.41	+	-
42	13.21	$C_{29}H_{30}O_8$	545.1592[M+K]+	545.1578	385.1644, 355.1539, 323.1277	Schisphenins G	-2.57	-	+
43	13.55*	$C_{20}H_{24}O_4$	328.1663[M]+	328.1675	295.1322, 236.9827, 192.9955, 176.9632, 137.0611	Anwulignan	3.66	-	+
44	14.05*	$C_{24}H_{32}O_6$	455.1816[M+K]+	455.1836	417.2272, 402.2019, 386.2089, 370.1782, 316.1317	Schisandrin A	4.39	+	+
45	14.59	$C_{27}H_{32}O_8$	523.1734[M+K]+	523.1734	385.1604, 370.1822	Schinlignans D	0.00	-	+
46	15.51	$C_{23}H_{28}O_6$	401.1974[M+H]+	401.1964	386.1727, 370.1782, 331.1152, 300.0973	Isomer of schisandrin B	-1.00	+	-
47	15.8	$C_{23}H_{28}O_6$	401.1974[M+H]+	401.1964	386.1727, 370.1782, 331.1152, 300.0973	Schisandrin B	-4.49	+	-
48	15.59	$C_{30}H_{40}O_4$	503.2554[M+K]+	503.2564	447.2907, 429.2797, 419.2896, 213.1256	Schisanlactone A	1.99	-	+
49	15.92	$C_{24}H_{30}O_6$	415.2105[M+H]+	415.212	384.1935, 369.1674, 338.1500	Methylgomisin O	3.61	-	+
50	16.2	$C_{30}H_{30}O_8$	557.1553[M+K]+	557.1578	397.1636, 367.1540, 351.1216	Schisanchinin A	4.49	-	+
51	16.72	$C_{28}H_{36}O_7$	485.2519[M+H]+	485.2539	385.1604, 368.1620, 353.1368	Schinlignans G	-0.41	+	-

52	16.81	$C_{22}H_{24}O_6$	385.1644[M+H]+	385.1651	355.1539, 315.0851, 285.0739, 257.0791, 227.0685	Schisandrin C	1.27	+	-
53	16.86	$C_{28}H_{34}O_8$	537.1886[M+K]+	537.1891	399.1800, 368.1620, 384.1574	Angeloylisogomisin O	0.93	-	+
54	17.31	$C_{30}H_{32}O_8$	543.1980[M+Na]+	543.1995	399.1800, 368.1620, 353.1368, 337.1426	6-O-Benzoylgomisin O	2.76	+	+
55	17.48	$C_{28}H_{34}O_8$	537.1886[M+K]+	537.1891	399.1800, 384.1534, 368.1620, 337.1426	Tigloylgomisin O	0.19	+	-
56	17.88	$C_{29}H_{28}O_8$	543.1408[M+K]+	543.1421	383.1477, 353.1368, 314.0801, 283.0605	Interiotherin A	2.39	-	+
57	18.08	$C_{28}H_{36}O_8$	539.2042[M+K]+	539.2047	383.1477, 368.1620	Micrantherin A	0.93	-	+
58	18.26	$C_{25}H_{34}O_7$	469.2233[M+Na]+	469.2203	386.1687, 354.1485, 325.1421	Wuweilignan E	-4.05	+	-

"+" indicates the presence of the compound, and "-" indicates the absence of the compound.

Figure Captions:

Figure S1. The total ion chromatogram from UPLC-Q-TOF/MS. (a) The total ion chromatogram of *S. chinensis*, Inner Mongolia (N13) as representative. (b) The total ion chromatogram of *S. sphenanthera*, Anhui (S15) as representative.

Figure S2. The total ion chromatogram from GC-MS. (a) The total ion chromatogram of *S. chinensis*, Inner Mongolia (N13) as representative. (b) The total ion chromatogram of *S. sphenanthera*, Anhui (S15) as representative.

Figure S3. The key antioxidant compounds in *S. sphenanthera.*

Figure S4. The key antioxidant compounds in *S. chinensis.*

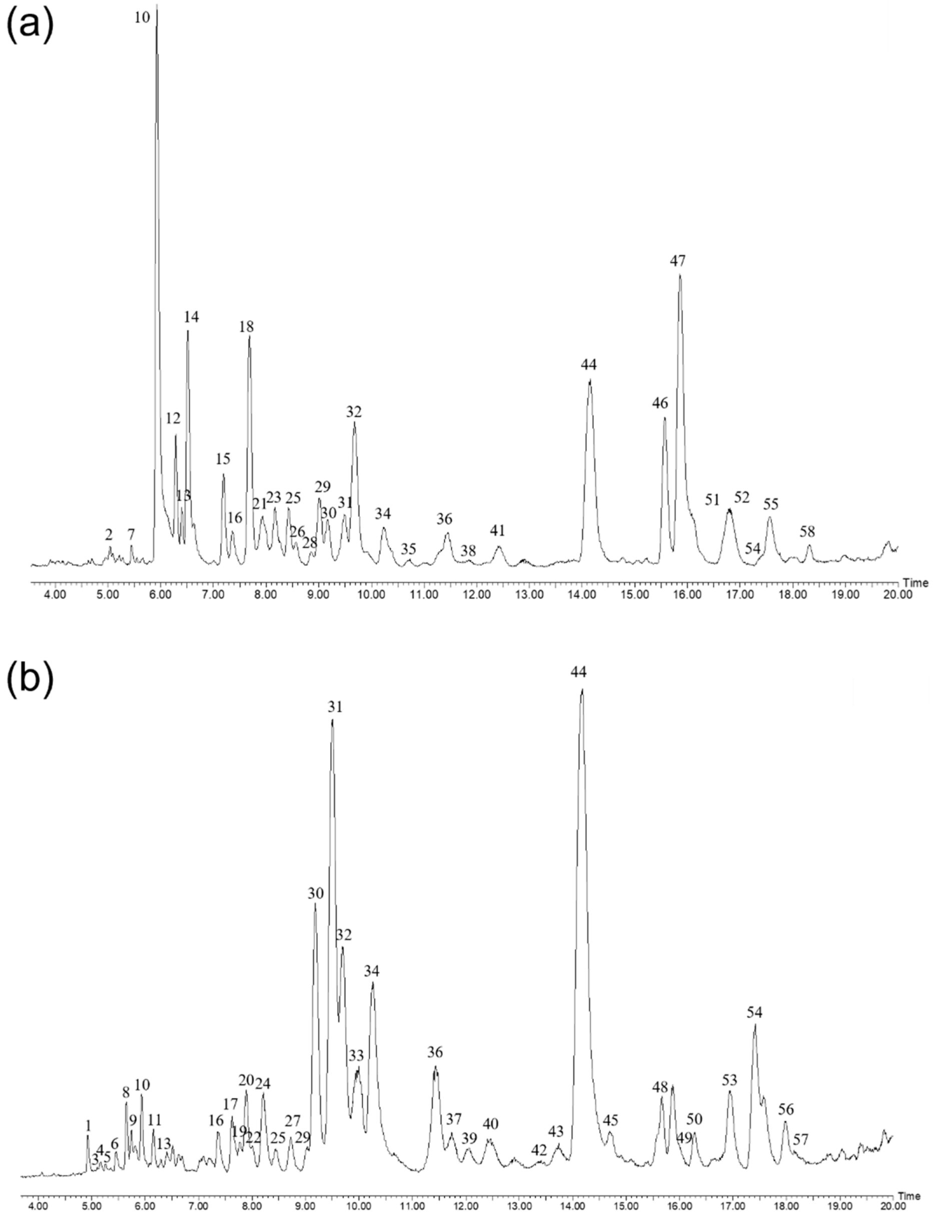

Figure S1. The total ion chromatogram from UPLC-Q-TOF/MS. (a) The total ion chromatogram of *S. chinensis*, Inner Mongolia (N13) as representative. (b) The total ion chromatogram of *S. sphenanthera*, Anhui (S15) as representative.

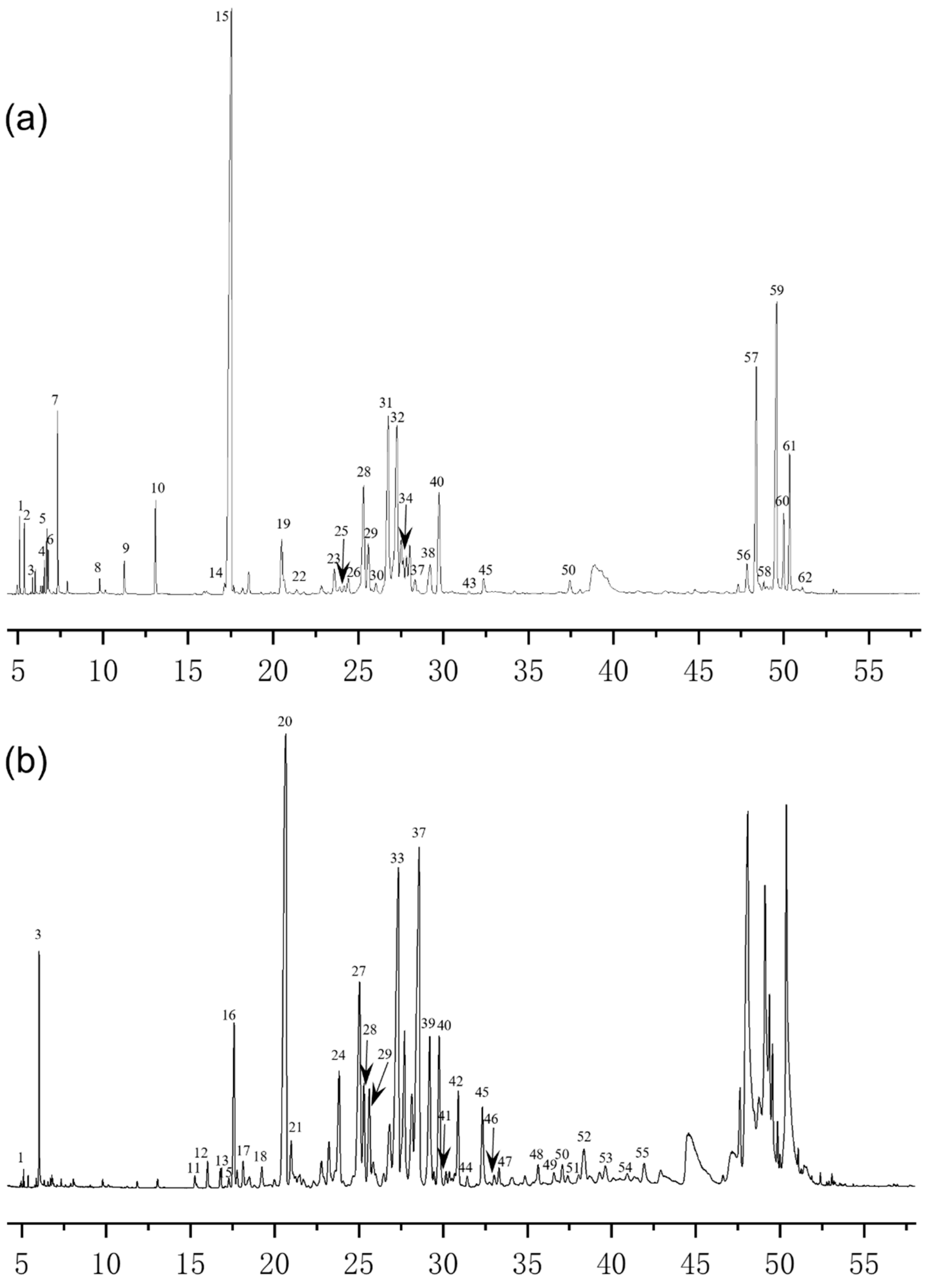

Figure S2. The total ion chromatogram from GC-MS. (a) The total ion chromatogram of *S. chinensis*, Inner Mongolia (N13) as representative. (b) The total ion chromatogram of *S. sphenanthera*, Anhui (S15) as representative.

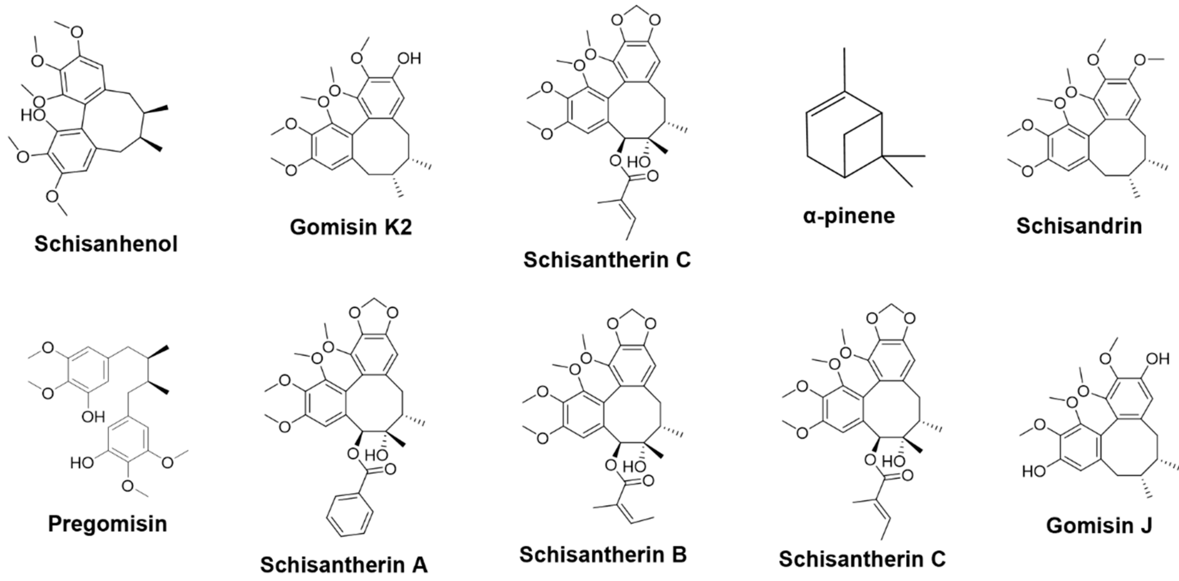

Figure S3. The key antioxidant compounds in *S. sphenanthera.*

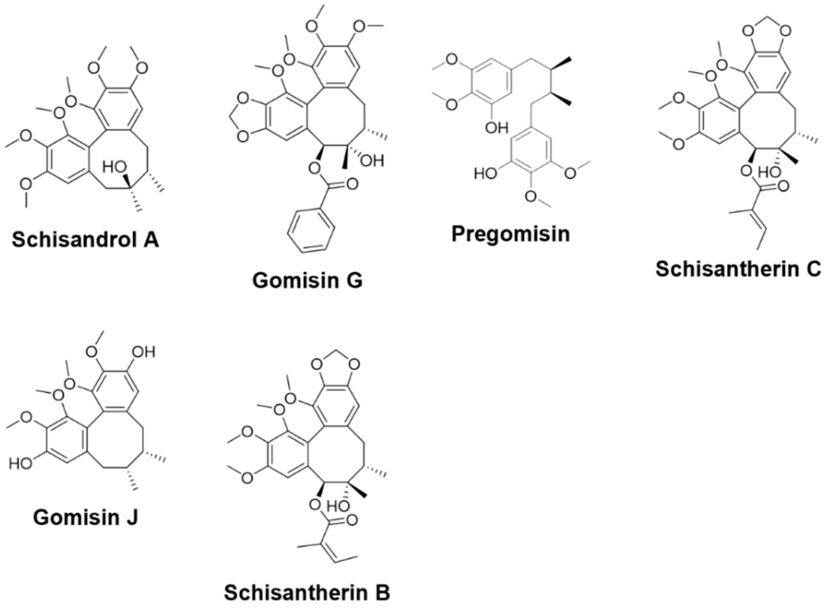

Figure S4. The key antioxidant compounds in *S. chinensis.*
